# Bioresponsive antisense DNA gold nanobeacons as a hybrid *in vivo* theranostics platform for the inhibition of cancer cells and metastasis

**DOI:** 10.1038/srep12297

**Published:** 2015-07-20

**Authors:** Chenchen Bao, João Conde, James Curtin, Natalie Artzi, Furong Tian, Daxiang Cui

**Affiliations:** 1 Institute of Nano Biomedicine and Engineering, Key Lab. of Thin Film and Microfabrication Technology of Ministry of Education, Department of instrument science and engineering, School of Electronic Information and Electrical Engineering, National Center for Translational Medicine, Shanghai Jiao Tong University, P.R.China; 2 Massachusetts Institute of Technology, Institute for Medical Engineering and Science, Harvard-MIT Division for Health Sciences and Technology, Cambridge, Massachusetts, USA; 3 School of Engineering and Materials Science, Queen Mary University of London, London, UK; 4 School of Food Science and Environmental Health, College of Sciences and Health, Dublin Institute of Technology, Cathal Brugha Street, Dublin, Ireland; 5 Department of Anesthesiology, Brigham and Women’s Hospital, Harvard Medical School, Boston, Massachusetts, USA; 6 Focas Research Institute, Dublin Institute of Technology, Camden Row, Dublin, Ireland

## Abstract

Gold nanobeacons can be used as a powerful tool for cancer theranostics. Here, we proposed a nanomaterial platform based on gold nanobeacons to detect, target and inhibit the expression of a mutant *Kras* gene in an *in vivo* murine gastric cancer model. The conjugation of fluorescently-labeled antisense DNA hairpin oligonucleotides to the surface of gold nanoparticles enables using their localized surface plasmon resonance properties to directly track the delivery to the primary gastric tumor and to lung metastatic sites. The fluorescently labeled nanobeacons reports on the interaction with the target as the fluorescent Cy3 signal is quenched by the gold nanoparticle and only emit light following conjugation to the *Kras* target owing to reorganization and opening of the nanobeacons, thus increasing the distance between the dye and the quencher. The systemic administration of the anti-Kras nanobeacons resulted in approximately 60% tumor size reduction and a 90% reduction in tumor vascularization. More important, the inhibition of the Kras gene expression in gastric tumors prevents the occurrence of metastasis to lung (80% reduction), increasing mice survival in more than 85%. Our developed platform can be easily adjusted to hybridize with any specific target and provide facile diagnosis and treatment for neoplastic diseases.

Amolecular beacon is a hairpin DNA single-stranded oligonucleotide, which transports a dye and a quencher at both ends. In the absence of a complementary target, the stem-loop structure is closed imposing the dye and the quencher to a close proximity, causing a fluorescence quenching. In the presence of a complementary target, the stem-loop sequence is opened and the molecular beacon hybridizes, creating a double-stranded 3D structure. With the opening of the stem-loop, the spatial distance between dye and quencher increases and the fluorescence is reestablished^1^.

Molecular beacons can be optimized to match with specific experimental requirements. By controlling the length of both stem and loop a broad range of temperatures for target detection can be achieved, as well as lower signal-to-background ratios and/or different dissociation rate constants^2^. Although very productive for *in vitro* experiments, utilization of molecular beacons *ex vivo* and *in vivo* has not been successful, probably due to the reduced chemical stability of nucleic acids in biological media and fragile protection against serum nucleases.

The growing field of nanotechnology has brought a wide variety of tools for molecular analysis and clinical imaging and theranostics with increased sensitivity and specificity^3^,^4^,^5^,^6^,^7^,^8^,^9^. Due to their optical properties, nanomaterials and nanoparticles in particular have been used for nucleic acid screening methodologies, specifically through thiolated DNA oligonucleotides capable of recognizing specific nucleotide sequences^10^,^11^,^12^,^13^. The combination of the optical properties of nanoparticles with the demonstrated advantages of standard molecular beacons can prove to be extremely useful in molecular biology. Gold nanoparticles (AuNPs) have been demonstrated to modulate dye emission in their vicinity^14^, from fluorescence quenching to fluorescence enhancement^15^, which suggests AuNPs as an excellent substitute of classic quenchers. Additionally, AuNPs provide the possibility for *in vivo* studies of DNA hairpin-like structures as they offer protection against degradation by nucleases^16^,^17^ and can act as both transfection and targeting vectors^18^,^19^,^20^ controlling crucial cellular processes such as antisense DNA and RNA interference pathways.

Antisense DNA^21^,^22^ pathways have emerged as powerful and useful tools to block gene function and for sequence-specific posttranscriptional gene silencing. The discovery of their prominent role in regulation of specific gene expression in numerous disease states, such as cancer resulted into an enormous effort towards the development of new efficient delivery systems that control those pathways^23^,^24^.

Gastric cancer (GC) is a prevalent cancer in Asia, with most new cases and deaths in China, Japan and Korea. It is the third most common malignant tumor and causes 300,000 deaths in China each year^25^. The 5-year survival rate for patients with advanced GC is only around 10%; the rest of the patients usually have median survival times of 8–12 months^26^. Improving the survival of GC patients requires the development of novel diagnostic and anti-tumor therapy. A *Kras* mutation have recently been described in gastrointestinal stromal tumors (GISTs), yielded a high number of KIT mutant GISTs and reported also in wild type for KIT and PDGFRA^27^,^28^.

*Kras* is one of the most commonly activated oncogenes in human cancer, with 17 to 25% of all human tumors harboring an activating *Kras* mutation^29^ In addition to gastrointestinal tumors, over 90% of pancreatic adenocarcinomas, 30%–50% of colorectal cancers, 55% of thyroid cancers, 35% of lung cancers, and 35% of rhabdomyosarcomas harbor mutated RAS genes^30^.

Previously, Conde *et al.* developed an *in vitro* gold-nanobeacon system as a proof-of-concept to follow RNA synthesis in real time in bulky solutions and for antisense DNA and RNA interference (RNAi), from gene specific silencing to silence-the-silencers *in vitro* only^31^,^32^,^33^. From these studies, the authors proved the potential of a single molecular nanoconjugate to intersect all RNA pathways *in vitro*: from gene specific downregulation (ie. inhibit a GFP reporter) to silencing siRNA and miRNA pathways^31^.

Here, we used antisense DNA gold nanobeacons to selective inhibition of mutant Kras *in vivo*. These bioresponsive gold nanobeacons have a unique and positive effect on metastatic cell colonization in the lungs in a syngeneic and spontaneous gastric cancer mice model. This nanobeacons platform is of high therapeutic potential and serves as a theranostics system of orthotopic gastric tumors but also as anti-metastasis treatment, where nanobeacons show a strong involvement in both local and distant lesions.

Therefore, we designed and developed an *in vivo* universal tool based on AuNPs functionalized with a dye labeled hairpin-DNA, i.e. gold-nanobeacon to inhibit simultaneously cancer cells and metastasis in a murine tumor model following systemic administration via the antisense silencing of a mutant transcript of *Kras* gene (see Fig. 1A). The NPs used in this study consist of a AuNP core decorated with and a thiol-DNA-hairpin labelled with a Cy3 dye. A polyethylene glycol (PEG) is also used as a spacer between the antisense DNA hairpins, increasing the nanostructure stability in biological medium^32^,^33^ (see Supplementary Figure S1 for PEG optimization and quantification). Under hairpin configuration, the proximity of the Cy3 dye to the AuNP (that works as quencher) leads to fluorescence quenching. Hybridization of the DNA hairpin to a complementary target (i.e. Kras mRNA) restores fluorescence emission due to the gold nanobeacons’ conformational reorganization that causes the fluorophore and the quencher to part from each other, yielding a quantitative response. These molecular beacons have the ability to interlock or hybridize with the target *Kras* mRNA (blocking specific translation initiation signals of *Kras* gene) thus inhibiting the translation of the target protein.

In addition to designing anti-Kras nanobeacon that detects and inhibits *Kras* mRNA, a nonsense nanobeacon (that does not hybridize with any target) was developed as an internal control (Supplementary Figure S2 and Table S1). Oligomers sequences and 2-dimensional structures of the beacons at 37 °C are depicted in Supplementary Table S1.

Transmission electron microscopy (TEM) images of the DNA antisense gold nanobeacons anti-Kras showed an average diameter of the gold core of 15.1 ± 1.1 nm (Supplementary Figure S3A). The final beacon:NP ratio was around 45:1 (see Supplementary Table S2). The mean particle diameter of nanobeacons anti-Kras is 25.9 (±3.5) nm and nanobeacon nonsense is 24.7 (±2.9) nm as measured by DLS and with an SPR peak at 525 nm as evaluated by UV-Vis extinction profiles (Supplementary Figure S3B), and slightly anionic (−25.6±1.2 mV) via zeta-potential (Supplementary Table S2).

The stability of gold nanobeacons towards increasing concentrations of Glutathione (GSH) and DNase was also evaluated (Supplementary Figure S4A,B). These data confirms the stability of the gold nanobeacons to intracellular concentrations of GSH (Supplementary Figure S4A) and DNase (Supplementary Figure S4B).

The fluorescent emission from the Cy3 (Emi = 570 nm) is efficiently quenched by the ~14 nm AuNP due to a nanosurface energy transfer (NSET) effect^34^. The fluorescence is “OFF” when the hairpin-DNA-Cy3 is closed (i.e. no hybridization with the complementary target). The fluorescence is “ON” when the hairpin-DNA-Cy3 hybridizes with a *Kras* complementary target and an increase in Cy3 emission occurs (Supplementary Figure S5). It is worth noting that the fluorescence emission intensity of Cy3-labeled DNA oligo is significantly decreased after the reaction with gold nanoparticles, due to the quenching effect between the Cy3 dye and the gold surface. This effect occurs once a good overlap between dye emission and quencher (AuNP) absorbance is observed, providing an indication of the quenching efficiency (Supplementary Figure S6A). When the concentration of Cy3-labeled DNA oligo was fixed at 1 μM, the increase in AuNPs concentration from 0 to 8 nM in the reaction mixture resulted in a significant increase in Cy3 fluorescence quenching (Supplementary Figure S6B), demonstrating the presence of NSET between the Cy3 groups and the AuNPs. These data showed a quenching efficiency of nearly 90% when the concentration of AuNPs reached 3 nM, whereas nearly 100% when the concentration of AuNPs reached 8 nM. Conversely, the Cy3 emission can be restored after hybridization with the complementary ssDNA Kras target. Increasing amounts of complementary target results in elevated emission form the Cy3 dye at 570 nm until reaching a plateau at 1E–05M of target (Fig. 1B).

Indeed, analytical flow cytometry data confirmed the signal specificity of the nanobeacon incubated in human gastric cancer cells (MGC-803), where a substantial increase in Cy3 intensity signal is observed only for nanobeacons anti-Kras when compared to nanobeacon nonsense (Fig. 1C).

We next assessed *in vitro* gene silencing efficiency in the human gastric cancer cell line MGC-803 of both anti-Kras and nonsense nanobeacons. The ability of both anti-Kras and nonsense nanobeacons to silence Kras was evaluated in gastric cancer cells, where only the anti-Kras nanobeacons provided a robust knockdown in a dose dependent manner, with a median effective dose (ED_50_) between 1 and 5 nM. Specifically, more than 80% silencing could be observed at the mRNA level by qPCR at a dose of 50 nM at 48 hours of incubation (Fig. 1D).

In order to corroborate flow cytometry data (Fig. 1C) the fluorescence signal from gold nanobeacons previously incubated with a ssRNA Kras target (mimicking Kras mRNA) was evaluated by the live imaging system (Fig. 1E). As expected, only anti-Kras nanobeacons produce a significant increase in fluorescence signal, when compared to nonsense nanobeacons and a blank.

The efficiency of the anti-Kras nanobeacon probes in sensing and in silencing gastric cancer cells *in vivo* was evaluated in an orthotopic gastric cancer mice model. Human gastric cancer cell line MGC-803 was used to generate the orthotopic tumor model in nude mice. After the formation of the tumor tissue, the tumor tissue stability in nude mice was passaged three more generations in subcutaneous tumors, before transferred into nude stomach via small animal surgery. An OB adhesive construction of orthotopic gastric tumor transplantation was applied for tumor tissue adhesion. OB glue paste technique is commonly used to establish nude mouse human gastric cancer orthotopic transplantation models. The OB glue paste technique is easy to perform and the biological behaviors of the nude mice human gastric cancer orthotopic transplantation models established with this technique are similar to the natural processes of growth and metastasis of human gastric cancer^35^. Four weeks after the OB surgery and the establishment of the orthotopic model, mice were tail-vein injected with gold nanobeacons and *in vivo* imaging was used to track simultaneously organs biodistribution, tumor size and nanobeacon probes signal after hybridization to the target monitored by fluorescence emission over a period of 120 hours (5 days) following nanobeacons injection (Fig. 2). The trial endpoint was 5 days once after that the signal from the nanobeacons decreased dramatically. *In vivo* imaging of mice administered with 5 or 50 nM of anti-Kras nanobeacons or nonsense nanobeacons (50 nM) revealed that only nanobeacons anti-Kras were able to promote efficient and sustained detection of primary gastric tumor (Fig. 2A,B), with an approximately 60% tumor size reduction 5 days after intravenous injection, when compared to nonsense nanobeacon. Concerning nanobeacon probes imaging, fluorescence images of treated mice revealed, as expected, that fluorescence signal is OFF at day 0 (0.5 hours after tail-vein injection) and is turned ON 4 hours later, and maintains the signal until 120 hours (Fig. 2A), only for the anti-Kras nanobeacons and not for the nonsense nanobeacons.

Recently, a similar system using gold nanobeacons for both gene and drug delivery in a multidrug resistance mice model was described^36^. The authors reported that gold nanobeacons could enter cells probably via an endocythosis-mediated mechanism. Confocal images of breast cancer cells further revealed that the gold nanobeacons were colocalized in intracellular organelles such as endosomes and/or lysosomes at 24 hours and could escape out of this organelles at 48 hours. If the majority of gold nanobeacons escape lysosome the mechanism behind the cellular uptake and endosomal escape is probably an endocytosis mechanism mediated by caveolae receptors, once the escape from the endosomal compartments happens before lysosomal activation. This mechanism represents an alternative pathway with distinct cellular compartments to avoid lysosomal degradation. The anionic nature of the nanobeacons may be endocytosed through the interaction with the positive site of the proteins in membrane, and they can be highly captured by cells because of their repulsive interactions with the negatively charged cell surface; and are more likely to use caveolae-dependent endocytosis.

Representative images of whole body organs and resected tumors in mice treated with 5 and 50 nM of anti-Kras nanobeacons is depicted in Fig. 2B. Other organs showed residual accumulation, consistent with previous work reported for AuNPs^28^. These results showed that anti-Kras nanobeacons accumulate also in lungs. As evaluated by the quantification of nanobeacons signal the anti-Kras nanobeacons at 5 and 50 nM accumulate preferentially at the gastric tumor site and lungs (Fig. 2C). Quantification analysis of tumor ROI also revealed the specificity of the anti-Kras nanobeacons to the gastric tumors, when compared to nonsense nanobeacons (Supplementary Figure S7). Moreover, monitoring tumor ROI as a function of time after treatment revealed a significant tumor size reduction using 50 nM of anti-Kras nanobeacons, when compared with 5 nM.

It is well known that metastasis occur frequently from gastric tumors to the lungs^37^. Therefore, in order to track gold nanobeacons in tumor and lung tissues plasmonic-tunable Raman/FITR imaging spectroscopy was used (Fig. 3). Figure 3A shows the FTIR image of gold nanoparticles only. The cancer tissue was under processed with standard cryosection steps using regular histopathology methodologies. The sections were spread on 2 mm CaF2 substrate and remained embedded in paraffin, without any chemical treatment such as dewaxing. The reason for this is to prevent any further chemical modification of the sample due to reagents and also to create some degree of refractive index matching, thus reducing the spectral distortion due to Mie scattering^38^.

At the spectral region with frequencies 750–4000 cm^−1^, absorption was more intense for the anti-Kras nanobeacons treated mice. The spectrum extracted from the tumor region shows absorption peaks at 1712, 1662, 1493 and 1082 cm^−1^, representing the DNA phosphate backbone stretching vibrations of anti-Kras nanobeacons (Fig. 3E), while the spectrum extracted from the tumor region with nonsense nanobeacons shows weaker bands at 1712, 1662, 1493 and 1082 cm^−1^. The infrared band at 1712 cm^−1^ exemplifies the H-bonded C2 = O2 stretching vibration of thymines (T) from the third strand in T*A–T triplex, where the third strand is involved in a reverse Hoogsteen base pair scheme. The band at 1662 cm^−1^ is attributed primarily to thymine stretching vibrations of C4 = O4 bonds. The band at 1493 cm^−1^ appears due to the ring stretching vibrations of cytosine (C = C) respectively^39^,^40^,^41^,^42^. In FTIR spectra of nonsense nanobeacons treated mice a new peak at 1750 cm^−1^ appears and the peak at 1082 cm^−1^ (that corresponds to the vibration intensity of the phosphoric acid groups (PO^2−^) with a symmetric stretching at, which has been extensively reported for several other material types^43^) is shifted to 1100 cm^−1^. Therefore, for the PO^2−^ symmetric stretching frequency, the band from the anti-Kras nanobeacons treated mice exhibit a peak at 1082 cm^−1^ in the original profile (Fig. 3E), and the peak from the nonsense nanobeacons treated mice appeared at 1100 cm^−1^ (Fig. 3C). Higher spectra peaks can be observed in anti-Kras nanobeacons when compared to nonsense nanobeacon, probably due to higher values of free energy in secondary structure formation (Supplementary Figure S2).

Spatial variations in the absorbance of these bands indicate the ability of micro ATR-FTIR imaging to probe chemical differences in tumor area (Fig. 3B,D). In addition to biological tissue, macro ATR-FTIR imaging has also been applied to study biomaterial processes including the plasmonic-tunable Raman/FITR imaging spectroscopy, which results show that anti-Kras nanobeacons accumulate extensively in the tumor tissue when compared to the nonsense nanobeacons.

Nevertheless, both nanobeacons accumulate similarly in lung tissue (Fig. 4A,B). In order to validate these data, inductively coupled plasma mass spectrometry (ICP-MS) showed that after 5 days of treatment, only anti-Kras nanobeacons accumulate extensively in tumor tissue; whereas both anti-Kras and nonsense nanobeacons accumulate in lung tissue (with a 5-fold increase when compared to other organs, such as heart, liver, spleen, kidney – Fig. 4C). Other organs showed residual accumulation, consistent with previous work reported for AuNPs^44^.

The higher gold content in the tumor tissue by the anti-Kras nanobeacons may be due to a delay in nanoparticles recycling by the cells, once the anti-Kras nanobeacons may stay bound to the target mRNA. Once the nonsense nanobeacons do not present any function within the cell they excreted by the cell much faster than the anti-Kras nanobeacons. This fact is corroborated by the data on the nanobeacons’ *in vitro* uptake kinetics measured by ICP-MS (see Supplementary Figure S8).

To validate safety assessment, 5 days after injection with nanobeacons organs were harvested from mice and H&E stained for routine pathological analysis (Supplementary Figure S9). H&E staining showed that, *in vivo* administration of both nonsense and anti-Kras nanobeacons did not cause any significant damage in several organs (i.e. heart, liver, spleen, and kidney).

However, histological analysis in tumor tissue showed evidence of extensive reduced vascularization for anti-Kras nanobeacons loaded when compared to nonsense nanobeacons (Fig. 5A and Supplementary Figure S9), in accordance with tumor size reduction (see Fig. 2B). Moreover, H&E staining of lung tissue show that nonsense nanobeacon exposed groups exhibited inflammatory accumulation at the bronchoalveolar junction and thickened local alveolar walls (Fig. 5A and Supplementary Figure S9). Significant reduction in severe interstitial infiltration of inflammatory cells is noted in the anti-Kras nanobeacon treated group. Nonsense nanobeacon treated group showed a predominance of mononuclear cells, as well as perivascular and peribronchiolar edema, occurrence of hypercellularity and thickened local alveolar walls and septa and increased number of tumoral clones (arrows in Fig. 5A). These histological images confirmed an evident decrease (~90%) in the incidence and severity of tumor clones and metastasis in lung from mice treated with anti-Kras nanobeacons, after 5 days of exposure.

Previous studies have been shown that metastatic cells could be established in the lungs before their oncogenic transformation, forming intravascular colonies in the lungs with concomitant invasion of the organ^45^,^46^. In fact, the incidence of pulmonary metastatic lung cancer from gastric cancer has been reported as approximately 50%, based on autopsy results^36^,^47^.

In order to confirm the reduction in number of gastric cancer metastasis into lung tissue, immunocolocalization staining’s were performed. Representative confocal microscopy images of lung tissue, confirmed the persistence of gastric tumor cells presence in the lungs (Fig. 5B) only in the group treated with nonsense nanobeacons. Alexa-Fluor®594-labeled anti-human vimentin and FITC-labeled anti-human HLA antibodies specific for human antigens (that do not cross-react with either mouse fibroblasts or tumor cells) were used to visualize human gastric cancer metastasis in lung from mice treated with nanobeacons, as described elsewhere^48^. Vimentin is a type III intermediate filament (IF) protein that is expressed in mesenchymal cells, and is often used as a marker of mesenchymal-derived cells or cells undergoing an epithelial-to-mesenchymal transition (EMT) during both normal development and metastatic progression^49^. On the other hand, the human leucocyte antigen (HLA) system is the major histocompatibility complex in humans, playing an essential role in the immune recognition of tumor cells that escape the immune system, and lead to lung metastasis for example^50^.

Vimentin and HLA expression is much higher in lung tissue treated with nonsense nanobeacons, when compared to anti-Kras nanobeacons (Fig. 5B). These results corroborate the decrease in the number of gastric cancer metastasis in lung tissue (~80% decrease) via the administration of anti-Kras nanobeacons (Fig. 5C).

Next, we hypothesized that anti-Kras nanobeacons would enhance the likelihood of observing an increase in mice survival, as their administration results in significant tumor size reduction and in the decrease of number of metastatic lung cells. Accordingly, nude mice bearing human gastric xenograft tumors were treated with both nonsense and anti-Kras nanobeacons (50 nM). Animals treated with anti-Kras nanobeacons survived significantly longer (***P < 0.005 – Fig. 5D) compared to nonsense nanobeacon group. The results indicated that the observed survival extension of more than 85% could be attributed to both the inhibition of gastric tumor cells in the primary tumor and by the extensive reduction in the occurrence of metastasis in lung. The improved outcome in tumor-bearing mice receiving anti-Kras nanobeacon treatment strongly supports the extraordinary potential of these nanoparticles as adjuvant agents to anticancer therapies. Effectively targeting metastases has proven to be extremely challenging due to the difficulty in overcoming biological barriers surrounding the cancer cells and the lack of tissue specificity. These bioresponsive antisense DNA gold nanobeacons prove to be a successful platform to target both cancer cells and metastasis. Our developed platform can be easily adjusted to hybridize with any specific target and provide rapid and cheap diagnosis and treatment for neoplastic diseases.

Taken together, treatment with antisense DNA anti-Kras nanobeacons efficiently and selectively reduced the primary gastric tumor size by 60% and impaired the establishment of lung metastatic cells in more than 80% of the cases, thus increasing mice survival in more than 85%. Our data indicate that gastric tumor-associated cells shed from the primary tumor together with accompanying cancer cells survive to a secondary site and proliferate within the metastatic nodules in the lung in the absence of anti-Kras nanobeacons treatment. Following selective *Kras* inhibition via antisense DNA nanoprobes, these unique nanobeacons have a positive effect on metastatic cell colonization in the lungs in a syngeneic and spontaneous mice model. In summary, the nanobeacons platform is of high therapeutic potential and serves as a theranostics and neoadjuvant therapy of orthotopic gastric tumors but also as anti-metastasis treatment, where nanobeacons show a strong involvement in both local and distant lesions. This platform can be further designed to selectively and specifically silence any target gene to overcome a broad range of cancerous diseases.

## Methods

### Synthesis of citrate gold nanoparticles

Gold nanoparticles, with an average diameter of 13.2 ± 2.1 nm, were synthesized by the citrate reduction method described by Lee and Meisel^51^. Briefly, 225 mL of 1 mM hydrogen tetrachloroaureate (III) hydrate (Sigma) (88.61 mg) was solved in 500 ml of distilled water, heated, and stirred under reflux. When the solution boils, 25 mL of 38.8 mM sodium citrate dihydrate (Sigma) (285 mg) was added resulting in a red solution. The solution is kept under ebullition with vigorous stirring and protected from light for 30 minutes. After this, the solution is cooled down and kept protected from light. Citrate gold nanoparticles were characterized by Transmission Electron Microscopy (TEM) and UV-Vis molecular absorption spectra.

### Synthesis of PEGylated-Gold nanoparticles

Functionalization of PEGylated gold nanoparticles was carried out using commercial hetero-functional poly(ethylene glycol) (PEG) functionalized with a 30% saturated surface of α-Mercapto-ω-carboxy PEG solution (HS-C_2_H_4_-CONH-PEG-O-C_3_H_6_-COOH, MW. 3500 Da, Sigma) as described elsewhere^52^,^53^. The 30% of saturated PEG layer allows the incorporation of additional thiolated components, such as the thiolated DNA-hairpin-Cy3. Briefly, 10 nM of the citrate-gold nanoparticles were mixed with 0.006 mg/mL of PEG solution in an aqueous solution of SDS (0.028%). After this, the mixture was incubated for 16 hours at room temperature. Excess PEG was removed by centrifugation (15,000 × rpm, 30 min, 4 °C), and quantified by a modification of the Ellman’s Assay (see Supplementary Information S2 for complete protocol and quantification methods).

### Synthesis of Gold Nanobeacons

Two different sequences of Gold Nanobeacons were prepared: a nanobeacon anti-Kras (detects and inhibits Kras mRNA) and a nanobeacon nonsense (designed to not hybridizes with any target within the genome) (Supplementary Table S1 for thiol-DNA-hairpin Cy3 sequences). Briefly, the thiolated oligonucleotides (Sigma) – thiol-DNA-hairpin Cy3 were suspended in 1 mL of 0.1 M dithiothreitol (DTT, Sigma), extracted three times with ethyl acetate and further purified through a desalting NAP-5 column (GE Healthcare) using 10 mM phosphate buffer (pH 8) as eluent. Following oligonucleotide quantification via UV/Vis spectroscopy, each oligomer was added to the AuNP-PEG solution in a 50:1 ratio. AGE I solution (2% (w/v) SDS, 10 mM phosphate buffer (pH 8)) was added to the mixture to achieve a final concentration of 10 mM phosphate buffer (pH 8), 0.01% (w/v) SDS. The solution was sonicated for 10 seconds using an ultrasound bath and incubated at room temperature for 20 minutes. Afterwards, the ionic strength of the solution was increased sequentially in 50 mM NaCl increments by adding the required volume of AGE II solution (1.5 M NaCl, 0.01% (w/v) SDS, 10 mM phosphate buffer (pH 8)) up to a final concentration of 10 mM phosphate buffer (pH 8), 0.3 M NaCl, 0.01% (w/v) SDS. After each increment, the solution was sonicated for 10 seconds and incubated at room temperature for 20 minutes before the next increment. Following the last addition, the solution was left to rest for additional 16 hours at room temperature. Then, the functionalized gold nanobeacons were centrifuged for 20 minutes at 15,000 × rpm, the oily precipitate washed three times with MilliQ water, and redispersed in MilliQ water. The resulting gold nanobeacons were stored in the dark at 4 °C until further use. Characterization of the gold nanobeacons was performed by Dynamic Light Scattering – DLS (Wyatt Dyna Pro Plate Reader), UV/Vis Spectroscopy and TEM (see Supplementary Table S2).

### Specificity assays for Gold Nanobeacons

For the detection of specific targets, 2.5 nM of nanobeacon anti-Kras and nanobeacon nonsense in 10 mM of phosphate buffer pH 7 was added to 5 nM of complementary Kras target (Sigma). All measurements were performed in a microplate reader (Varioskan Flash Multimode Reader, Thermo Scientific) programmed to incubate the reactions for 120 min at 37 °C while recording the fluorescence intensity every 2 minutes at an excitation wavelength of 550 nm for Cy3-labeled gold nanobeacon.

### *In vitro* Gold Nanobeacon’s delivery

Human gastric cancer MGC-803 cells were grown in Dulbecco’s modified Eagle’s medium (DMEM, Invitrogen) supplemented with 4 mM glutamine, 10% heat inactivated fetal bovine serum (Invitrogen), 100 U/ml penicillin and 100 μg/ml streptomycin (Invitrogen) and maintained at 37 °C in 5% CO_2_. Cells were seeded at a density of 1 × 10^5^ cells/well in 24-well plates and grown for 24 hours prior to incubation of nanobeacons. On the day of incubation, the cells were approximately 50% confluent. For flow cytometry, data were acquired on FACS LSR Fortessa HTS-1 (BD Biosciences) flow cytometer.

### Quantitative PCR

Total RNA from Human gastric cancer cell line MGC-803 was extracted using RNeasy Plus Mini Kit (Qiagen) according to the manufacture’s protocol. cDNA was produced using High-Capacity cDNA Reverse Transcription Kit (Applied Biosystems) using 500 ng of total RNA. qRT-PCR was performed with Taqman probes FAM-MGB for Kras and GAPDH (Applied Biosystems). GAPDH was used as a reference gene. The reactions were processed using Light Cycler 480 II Real-time PCR machine (Roche) using TaqMan® Gene Expression Master Mix (Applied Biosystems) under the following cycling steps: 2 min at 50 °C for UNG activation; 10 min at 95 °C; 40 cycles at 95 °C for 15 s; 60 °C for 60 s. At least three independent repeats for each experiment were carried out. Gene expression was determined as a difference in fold after normalizing to the housekeeping gene GAPDH.

### Construction of orthotopic gastric cancer mice model

All animal experiments were approved by the Institutional Animal Care and Use Committee of Shanghai Jiao Tong University (NO.SYXK2007-0025). The animal experimentation methods were carried out in accordance with the approved and relevant guidelines and regulations. Nude mice (nu/nu, female, 18 g, 4 weeks old) were purchased from the Shanghai SLAC Laboratory animal Co. Ltd and housed in SPF grade animal center. Subcutaneous gastric cancer (GC) tissues at the exponential growth phase loaded by nude mice were resected aseptically. Necrotic tissues were cut away, and the remaining tumor tissues were scissor-minced into pieces around 1–2 mm in diameter in 4 °C Hanks’ balanced salt solution, and each piece was adjusted to be 30 mg with scissors. Before the surgery, all mice were fasting for at least 8 hours. Mice were anesthetized with 5% trichloraldehyde hydrate, with 4 μL/g weight and an incision was made through the left upper abdominal pararectal line and peritoneum. The stomach wall was carefully exposed, and a part of the serosal membrane, about 2 mm in diameter, in the middle of the greater curvature of the glandular stomach was mechanically scratched with ophthalmic knives. A tumor piece of 30 mg was then fixed on the injured site of the serosal surface with OB glue (Guang Zhou Baiyun Ltd, China). The stomach was then returned to the peritoneal cavity, and the abdominal wall and skin were closed with 9-0 ophthalmic sutures. The animals were kept on warm electric blanket at 37 °C until analepsia and kept separately in an SPF environment.

### FTIR Instrumentation

ATR spectra were recorded with the Perkin Elmer Spotlight 400 N Universal Attenuated Total Reflectance (UATR) accessory of the spectrometer, which employs a 9-bounce diamond top-plate for this analysis. Sample penetration is both wavenumber and sample dependent, but is typically of at the order of 1 mm. In ATR mode, spectral data were the result of 4 scans, with a spectral resolution of 8 cm^**−**1^. Tissue samples were seed on CaF2 crystal and air dry for 10 min prior to recording. A background spectrum was also recorded and automatically subtracted by the software.

### Analysis of tumor growth and mice survival

Non-invasive longitudinal monitoring of tumor progression was followed by scanning mice with the Bruker *In-Vivo* F PRO imaging system from mice bearing gastric tumors (n = 6 animals per treated group). Whole-animal imaging was performed at the indicated time points – 0.5, 4, 24, 72 and 120 hours after nanobeacons injection. Kaplan-Meier survival curves were designed by evaluating mice survival follow up. In order to compare the survival distributions of mice treated with anti-Kras and nonsense nanobeacons log-rank tests were performed.

### Histology and immunolocalisation

After mice have been sacrificed and perfused with sterile PBS, organs were snap-frozen in optimum cutting temperature compound (Tissue-Tek®) and cut on a cryostat microtome and air-dried overnight. Frozen sections were thawed and stained for hematoxylin and eosin using standard procedures. Stained tissue sections were then imaged by light microscopy. For immunolocalisation, the tumor and lung tissues were embedded and sliced into a 3 μm section. After fixation with 4% paraformaldehyde in PBS, lung tissue sections were treated with blocking solution containing 4% rabbit serum in PBS. For metastasis detection, the lung tissue slide was incubated with Alexa Fluor® 594-labeled anti-human vimentin antibody (1:250 Abcam) and FITC-labeled anti-human HLA antibody (1:100, BioLegend) in PBS containing 1% BSA for 60 min, and was washed three times in PBS. Nuclei were DAPI stained before slide mounting with Fluoromount™ Aqueous Mounting Medium (Sigma). Stained tissue sections were then imaged by fluorescence microscopy.

### Biodistribution studies

quantification of gold nanobeacons accumulation in the tumor and other organs. Biodistribution of the functionalized gold nanobeacons in tissues associated with clearance (liver, spleen and kidney) as well as the gastric tumor, lung and heart was measured by inductively coupled plasma mass spectrometry (ICP-MS). Briefly, 5 days after gold nanobeacons administration, the mice were sacrificed to harvest the major organs, which were rinsed with ethanol three times and then air-dried into clean vials for acid digestion (*aqua-regia* 3HCl:1HNO_3_). After 1 day of strong acid digestion, the samples were analyzed by ICP-MS.

### Statistics

Differences between groups were examined using Student’s paired t test through SPSS statistical package (version 17, SPSS Inc.). All error bars used in this report are mean ± SD of at 3 independent experiments. Statistically significant P values were indicated in figures and/or legends as ***P < 0.005; **P < 0.01; *P < 0.05. All *in vivo* experiments used 5 mice per treatment group unless noted otherwise.

## Additional Information

**How to cite this article**: Bao, C. *et al.* Bioresponsive antisense DNA gold nanobeacons as a hybrid *in vivo* theranostics platform for the inhibition of cancer cells and metastasis. *Sci. Rep.* **5**, 12297; doi: 10.1038/srep12297 (2015).

## Supplementary Material

Supplementary Information

## Figures and Tables

**Figure 1:**
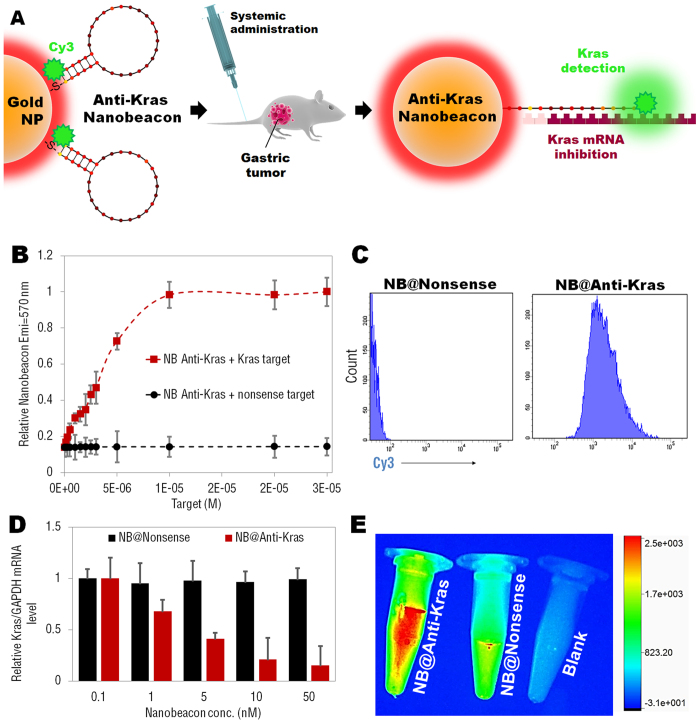
(**A**) Gold nanoparticles (AuNPs) functionalized with a dye (Cy3) labeled hairpin-DNA, i.e. gold-nanobeacon (Au-nanobeacon), designed to detect, target and inhibit the expression of *Kras* gene in an *in vivo* murine gastric cancer model. Under hairpin configuration, proximity to gold nanoparticles leads to fluorescence quenching; hybridization to a complementary target restores fluorescence emission due to the Au-nanobeacons’ conformational reorganization that causes the dye and the AuNP to part from each other. The scheme was designed and produced on *Adobe Illustrator CC 2014* by *J.Conde*. (**B**) Cy3 emission at 570 nm after hybridization with increasing amounts of complementary and non-complementary ssRNA targets (0 to 3E-05 M). (**C**) Flow cytometry analysis comparing nanobeacon anti-Kras treated cells to nanobeacon nonsense. (**D**) The silencing effect was expressed as a concentration-dependent decrease in *Kras* relative expression. Anti-Kras nanobeacons showed potent silencing effects in human gastric cancer cells (MGC-803) with an ED50 between 1 and 5 nM. (**E**) Live imaging images of anti-Kras nanobeacon (previously incubated with Kras complementary target) compared to nanonsense nanobeacon and a blank tube.

**Figure 2:**
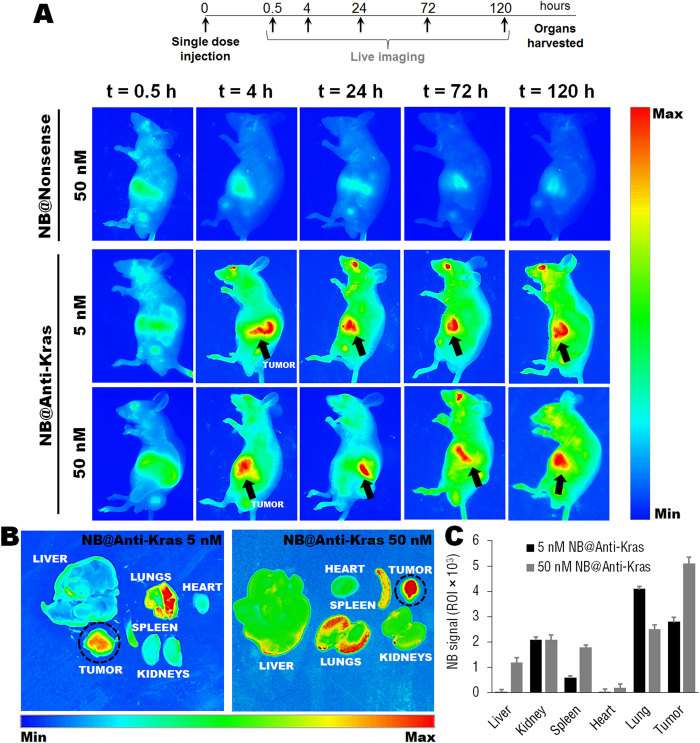
(**A**) Epi-fluorescence analysis of nude mice bearing orthotopic human gastric cancer cells, 0.5, 4, 24, 72 and 120 hours after tail-vein injection of nonsense (50 nM) and anti-Kras (5 and 50 nM) nanobeacons. Arrows represent gastric tumors. (**B**) Epi-fluorescence images of whole body organs to evaluate selective signal from the gold nanobeacons accumulated in tumors. (**C**) Quantification of anti-Kras nanobeacons signal in the region of interest (ROI) in major organs (liver, kidney, spleen, heart, lung and tumor) at 5 days after nanobeacons injection.

**Figure 3:**
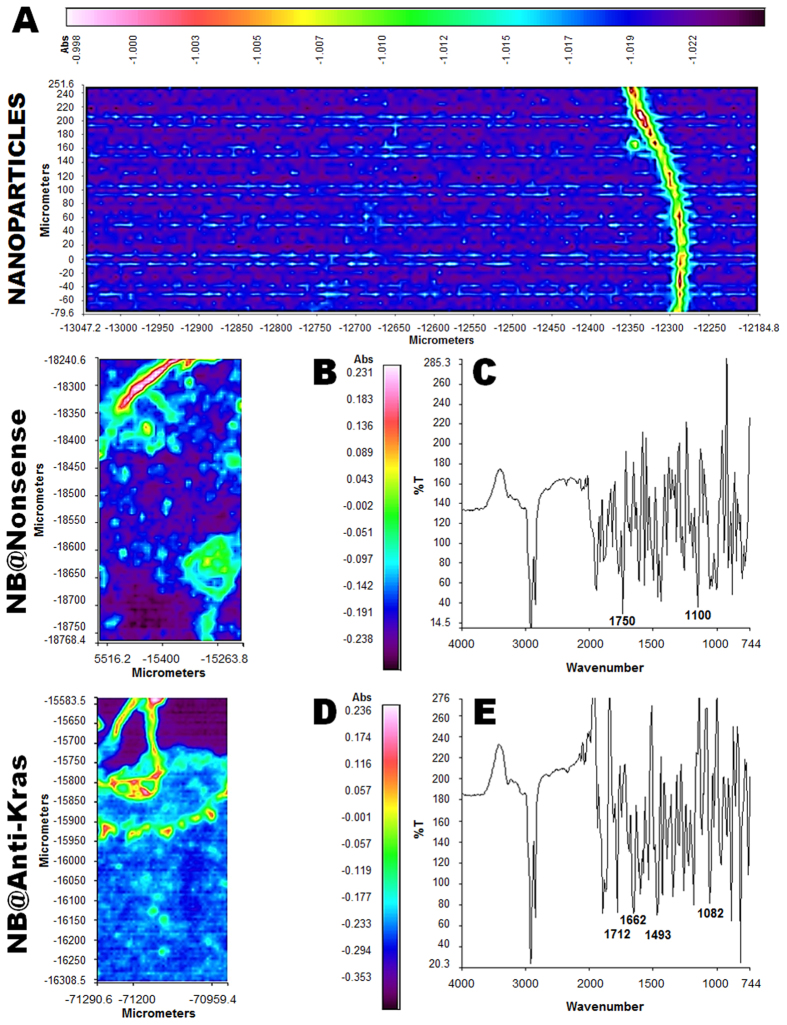
ATR-FTIR imaging of the tumor tissue for mice treated with naked nanoparticles (**A**), NB@nonsense (**B**) and NB@Anti-Kras (**D**) on tumor region. (**C**) The spectra extracted from the tumor region with nonsense nanobeacons weaker bands at 1712, 1662, 1493 cm^**−**1^ and new bands at 1750 and 1100 cm^**−**1^. (**E**) The spectra extracted from the tumor region of mice treated with anti-Kras nanobeacons show stronger bands at 1712, 1662, 1493 and 1082 cm^**−**1^.

**Figure 4:**
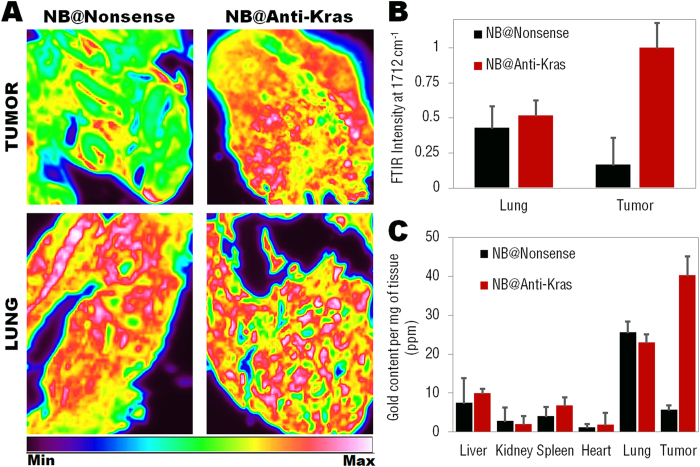
(**A**) ATR-FTIR image of nonsense and anti-Kras nanobeacons showing the integrated absorbance at 1712 cm^**−**1^ on tumor and lung area. (**B**) Relative ATR-FTIR intensity at 1712 cm^**−**1^ for nonsense and anti-Kras nanobeacons accumulated in tumor and lung tissues. (**C**) Quantification of gold accumulated in tissues by inductively coupled plasma mass spectrometry (ICP-MS). Biodistribution data of nonsense and anti-Kras nanobeacons in major organs (liver, spleen, lung, kidney, heart and tumor) at 5 days after nanobeacons injection. Note the difference in tumor accumulation between the nonsense and anti-Kras nanobeacons and in comparison with the other major organs. Both nonsense and anti-Kras nanobeacons accumulate significantly in lungs. The SD (error bars) were calculated based on six animals (n = 6) per each study group.

**Figure 5:**
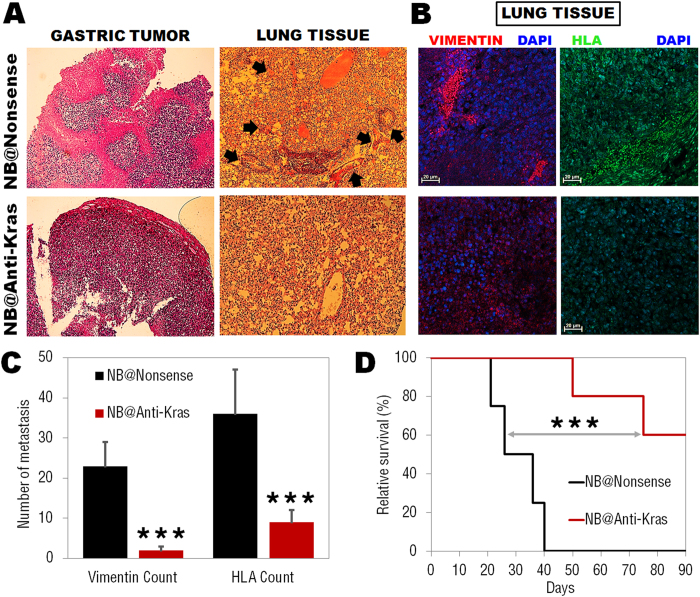
(**A**) Histological analysis of gastric tumor and lung tissue indicating a reduced vascularization in the primary tumor and a decrease in tumoral clones in lung tissue treated with anti-Kras nanobeacons only. Nonsense nanobeacons group present inflammatory cells accumulation at bronchoalveolar junction and thickened local alveolar walls and high prevalence of tumoral clones (arrows). (**B**) Representative confocal microscopy images of lung tissue immunostaining, confirming the persistence of gastric cancer metastasis in the lungs. Blue: DAPI nuclear stain; red: Alexa-Fluor®594-labeled anti-human vimentin; green: FITC-labeled anti-human HLA. Scale bars, 20 μm. (**C**) anti-Kras nanobeacons treatment significantly reduced the number of spontaneous lung metastasis in gastric cancer model (***P < 0.005, n = 6 mice). (**D**) Kaplan-Meier survival curves for mice treated with nonsense (black) and anti-Kras (red) nanobeacons using Log-Rank test. Data points represent group mean ± SD (n = 6, ***P < 0.005).

## References

[b1] Tyagi S. & Kramer F. R. Molecular beacons: probes that fluoresce upon hybridization. Nat. Biotechnol. 14, 303–308 (1996). 9630890 10.1038/nbt0396-303

[b2] Tsourkas A., Behlke M. A., Rose S. D. & Bao G. Hybridization kinetics and thermodynamics of molecular beacons. Nucleic Acids Res. 31, 1319–1330 (2003). 12582252 10.1093/nar/gkg212 PMC150230

[b3] Conde J., Bao C., Cui D., Baptista P. V. & Tian F. Antibody-drug gold nanoantennas with Raman spectroscopic fingerprints for *in vivo* tumour theranostics. J. Control Release 183, 87–93 (2014). 24704711 10.1016/j.jconrel.2014.03.045

[b4] Conde J., Doria G. & Baptista P. Noble metal nanoparticles applications in cancer. J. Drug Deliv. 2012, 751075 (2012). 22007307 10.1155/2012/751075 PMC3189598

[b5] Peer D. *et al.* Nanocarriers as an emerging platform for cancer therapy. Nat. Nanotechnol. 2, 751–760 (2007). 18654426 10.1038/nnano.2007.387

[b6] Palonpon A. F. *et al.* Raman and SERS microscopy for molecular imaging of live cells. Nat. Protoc. 8, 677–692 (2013). 23471112 10.1038/nprot.2013.030

[b7] Conde J. *et al.* RNAi-based glyconanoparticles trigger apoptotic pathways for *in vitro* and *in vivo* enhanced cancer-cell killing. Nanoscale 7, 9083–9091 (2015). 25924183 10.1039/c4nr05742b

[b8] Kumar S., Aaron J. & Sokolov K. Directional conjugation of antibodies to nanoparticles for synthesis of multiplexed optical contrast agents with both delivery and targeting moieties. Nat. Protoc. 3, 314–320 (2008). 18274533 10.1038/nprot.2008.1

[b9] Shu Y., Shu D., Haque F. & Guo P. Fabrication of pRNA nanoparticles to deliver therapeutic RNAs and bioactive compounds into tumor cells. Nat. Protoc. 8, 1635–1659 (2013). 23928498 10.1038/nprot.2013.097 PMC3883045

[b10] Conde J., De La Fuente J. M. & Baptista P. V. RNA quantification using gold nanoprobes - application to cancer diagnostics. J. Nanobiotechnology. 8, 5 (2010). 20181241 10.1186/1477-3155-8-5 PMC2844353

[b11] Zhang J., Song S., Wang L., Pan D. & Fan C. A. gold nanoparticle-based chronocoulometric DNA sensor for amplified detection of DNA. Nat. Protoc. 2, 2888–2895 (2007). 18007624 10.1038/nprot.2007.419

[b12] Liu J. & Lu Y. Preparation of aptamer-linked gold nanoparticle purple aggregates for colorimetric sensing of analytes. Nat. Protoc. 1, 246–252 (2006). 17406240 10.1038/nprot.2006.38

[b13] Cao Y. C., Jin R., Thaxton C. S. & Mirkin C. A. A two-color-change, nanoparticle-based method for DNA detection. Talanta 67, 449–455 (2005). 18970188 10.1016/j.talanta.2005.06.063

[b14] Rosa J. P., Lima J. C. & Baptista P. V. Experimental photophysical characterization of fluorophores in the vicinity of gold nanoparticles. Nanotechnology. 22, 415202 (2011). 21914933 10.1088/0957-4484/22/41/415202

[b15] Anger P., Bharadwaj P. & Novotny L. Enhancement and quenching of single-molecule fluorescence. Phys. Rev. Lett. 96, 113002 (2006). 16605818 10.1103/PhysRevLett.96.113002

[b16] Conde J., De La Fuente J. M., & Baptista P. V. *In vitro* transcription and translation inhibition via DNA functionalized gold nanoparticles. Nanotechnology. 21, 505101 (2010). 21098932 10.1088/0957-4484/21/50/505101

[b17] Rosi N. L. *et al.* Oligonucleotide-modified gold nanoparticles for intracellular gene regulation. Science 312, 1027–1030 (2006). 16709779 10.1126/science.1125559

[b18] Conde J. *et al.* *In vivo* tumor targeting via nanoparticle-mediated therapeutic siRNA coupled to inflammatory response in lung cancer mouse models. Biomaterials 34, 7744–7753 (2013). 23850099 10.1016/j.biomaterials.2013.06.041

[b19] Conde J. *et al.* Design of multifunctional gold nanoparticles for *in vitro* and *in vivo* gene silencing. ACS Nano. 6, 8316–8324 (2012). 22882598 10.1021/nn3030223

[b20] Patel P. C., Giljohann D. A., Seferos D. S. & Mirkin C. A. Peptide antisense nanoparticles. Proc. Natl. Acad. Sci. USA 105, 17222–17226 (2008). 19004812 10.1073/pnas.0801609105 PMC2582275

[b21] Modarresi F. *et al.* Inhibition of natural antisense transcripts *in vivo* results in gene-specific transcriptional upregulation. Nat. Biotechnol. 30, 453–459 (2012). 22446693 10.1038/nbt.2158 PMC4144683

[b22] Fichou Y. & Ferec C. The potential of oligonucleotides for therapeutic applications. Trends Biotechnol. 24, 563–570 (2006). 17045686 10.1016/j.tibtech.2006.10.003

[b23] Agrawal S. Antisense oligonucleotides: towards clinical trials. Trends Biotechnol. 14, 376–387 (1996). 8987636 10.1016/0167-7799(96)10053-6

[b24] Tamm I., Dorken B. & Hartmann G. Antisense therapy in oncology: new hope for an old idea? Lancet 358, 489–497 (2001). 11513935 10.1016/S0140-6736(01)05629-X

[b25] Jing J. J. *et al.* Gastric cancer incidence and mortality in Zhuanghe, China, between 2005 and 2010. World J. Gastroenterol. 18, 1262–1269 (2012). 22468091 10.3748/wjg.v18.i11.1262 PMC3309917

[b26] Meireles S. I. *et al.* Molecular classifiers for gastric cancer and nonmalignant diseases of the gastric mucosa. Cancer Res. 64, 1255–1265 (2004). 14973074 10.1158/0008-5472.can-03-1850

[b27] Hechtman J. F. *et al.* Novel oncogene and tumor suppressor mutations in KIT and PDGFRA wild type gastrointestinal stromal tumors revealed by next generation sequencing. Genes Chromosomes Cancer 54, 177–184 (2015). 25427437 10.1002/gcc.22230 PMC6452627

[b28] Qian Z. *et al.* Whole genome gene copy number profiling of gastric cancer identifies PAK1 and KRAS gene amplification as therapy targets. Genes Chromosomes Cancer 53, 883–894 (2014). 24935174 10.1002/gcc.22196

[b29] Kranenburg O. The KRAS oncogene: Past, present, and future. *Biochim*. Biophys. Acta. 1756, 81–82 (2005). 10.1016/j.bbcan.2005.10.001 16269215

[b30] Arrington A. K. *et al.* Prognostic and Predictive Roles of KRAS Mutation in Colorectal Cancer. Int. J. Mol. Sci. 13, 12153–12168 (2012). 23202889 10.3390/ijms131012153 PMC3497263

[b31] Rosa J., Conde J., De La Fuente J. M., Lima J. C., & Baptista P.V. Gold-nanobeacons for real-time monitoring of RNA synthesis. Biosens. Bioelectron. 36, 161–167 (2012). 22541812 10.1016/j.bios.2012.04.006

[b32] Conde J., Rosa J., De La Fuente J. M. & Baptista P. V. Gold-nanobeacons for simultaneous gene specific silencing and intracellular tracking of the silencing events. Biomaterials 34, 2516–2523 (2013). 23312904 10.1016/j.biomaterials.2012.12.015

[b33] Conde J. *et al.* Gold-nanobeacons for gene therapy: evaluation of genotoxicity, cell toxicity and proteome profiling analysis. Nanotoxicology 8, 521–532 (2013). 23642008 10.3109/17435390.2013.802821

[b34] Griffin J. *et al.* Size- and Distance-Dependent Nanoparticle Surface-Energy Transfer (NSET) Method for Selective Sensing of Hepatitis C Virus RNA. Chemistry 15, 342–351 (2009). 19035615 10.1002/chem.200801812

[b35] Shi J. *et al.* OB glue paste technique for establishing nude mouse human gastric cancer orthotopic transplantation models. World J. Gastroenterol. 14, 4800–4804 (2008). 18720543 10.3748/wjg.14.4800 PMC2739344

[b36] Conde J., Oliva N. & Artzi N. Implantable hydrogel embedded dark-gold nanoswitch as a theranostic probe to sense and overcome cancer multidrug resistance. Proc. Natl. Acad. Sci. USA 112, 1278–1287 (2015). 10.1073/pnas.1421229112 PMC4371927 25733851

[b37] Lee H. C. *et al.* Metastasis of gastric carcinoma to the thyroid and lung: a case report and review of literature. J. Zhejiang Univ. Sci. B. 11, 542–546 (2010). 20593521 10.1631/jzus.B0900378 PMC2897026

[b38] Bassan P. *et al.* Resonant Mie scattering in infrared spectroscopy of biological materials—understanding the ‘dispersion artefact’. Analyst 134, 1586–1593 (2009). 20448924 10.1039/b904808a

[b39] Banyay M., Sarkar M. & Graslund A. A library of IR bands of nucleic acids in solution. Biophys. Chem. 104, 477–488 (2003). 12878315 10.1016/s0301-4622(03)00035-8

[b40] Agarwal S., Jangir D. K., Singh P. & Mehrotra R. Spectroscopic analysis of the interaction of lomustine with calf thymus DNA. J. Photochem. Photobiol. B. 130, 281–286 (2014). 24368412 10.1016/j.jphotobiol.2013.11.017

[b41] Tyagi G., Pradhan S., Srivastava T. & Mehrotra R. Nucleic acid binding properties of allicin: Spectroscopic analysis and estimation of anti-tumor potential. Biochim. Biophys. Acta. 1840, 350–356 (2014). 24041991 10.1016/j.bbagen.2013.09.007

[b42] Jangir D. K., Kundu S. & Mehrotra R. Role of Minor Groove Width and Hydration Pattern on Amsacrine Interaction with DNA. Plos One 8, (2013). 10.1371/journal.pone.0069933 PMC3726726 23922861

[b43] Alex S. & Dupuis P. Ft-Ir and Raman Investigation of Cadmium Binding by DNA. Inorganica Chim. Acta. 157, 271–281 (1989).

[b44] De Jong W. H. *et al.* Particle size-dependent organ distribution of gold nanoparticles after intravenous administration. Biomaterials 29, 1912–1919 (2008). 18242692 10.1016/j.biomaterials.2007.12.037

[b45] Al-Mehdi A. B. *et al.* Intravascular origin of metastasis from the proliferation of endothelium-attached tumor cells: a new model for metastasis. Nat. Med. 6, 100–102 (2000). 10613833 10.1038/71429

[b46] Podsypanina K. *et al.* Seeding and propagation of untransformed mouse mammary cells in the lung. Science 321, 1841–1844 (2008). 18755941 10.1126/science.1161621 PMC2694414

[b47] Kong J. H. *et al.* Lung metastases in metastatic gastric cancer: pattern of lung metastases and clinical outcome. Gastric Cancer 15, 292–298 (2012). 22037917 10.1007/s10120-011-0104-7

[b48] Duda D. G. *et al.* Malignant cells facilitate lung metastasis by bringing their own soil. Proc. Natl. Acad. Sci. USA 107, 21677–21682 (2010). 21098274 10.1073/pnas.1016234107 PMC3003109

[b49] Dauphin M. *et al.* Vimentin expression predicts the occurrence of metastases in non small cell lung carcinomas. Lung Cancer 81, 117–122 (2013). 23562674 10.1016/j.lungcan.2013.03.011

[b50] He X. P. *et al.* The relationship between KRAS gene mutations and HLA class I antigen downregulation in the metastasis of non-small cell lung cancer. J. Int. Med. Res. 41, 1473–1483 (2013). 23975858 10.1177/0300060513489801

[b51] Lee P. C. & Meisel D. Adsorption and surface-enhanced Raman of dyes on silver and gold sols. J. Phys. Chem. 86 (17), 3391–3395 (1982).

[b52] Sanz V. *et al.* Effect of PEG biofunctional spacers and TAT peptide on dsRNA loading on gold nanoparticles. J. Nanop. Res. 14, (2012).

[b53] Conde J., Rosa J., De La Fuente J. M. & Baptista P. V. Gold-nanobeacons for simultaneous gene specific silencing and intracellular tracking of the silencing events. Biomaterials 34, 2516–2523 (2013). 23312904 10.1016/j.biomaterials.2012.12.015

