# Supplementary material for: Bioresponsive antisense DNA gold nanobeacons as a hybrid in vivo theranostics platform for the inhibition of cancer cells and metastasis
Source: Sci Rep. 2015 Jul 20;5:12297. doi: 10.1038/srep12297 (PMC4507177; doi:10.1038/srep12297)
Supplement: Supplementary Information [file srep12297-s1.pdf]

Supplementary Information

Bioresponsive antisense DNA gold nanobeacons as a hybrid *in vivo* theranostics platform for the inhibition of cancer cells and metastasis

Chenchen Bao[1,#], João Conde[2,3,#,*], Natalie Artzi[2,4], James Curtin[5], Furong Tian[6,*] and Daxiang Cui[1]

[1] Institute of Nano Biomedicine and Engineering, Key Lab. of Thin Film and Microfabrication Technology of Ministry of Education, Department of instrument science and engineering, School of Electronic Information and Electrical Engineering, National Center for Translational Medicine, Shanghai Jiao Tong University, P.R.China.

[2] Massachusetts Institute of Technology, Institute for Medical Engineering and Science, Harvard-MIT Division for Health Sciences and Technology, Cambridge, Massachusetts, USA.

[3] School of Engineering and Materials Science, Queen Mary University of London, London, UK.

[4] Department of Anesthesiology, Brigham and Women's Hospital, Harvard Medical School, Boston, Massachusetts, USA.

[5] School of Food Science and Environmental Health, College of Sciences and Health, Dublin Institute of Technology, Cathal Brugha Street, Dublin, Ireland.

[6] Focas Research Institute, Dublin Institute of Technology, Camden Row, Dublin, Ireland.

# These authors contributed equal.

*Corresponding authors, e-mail: furong.tian@dit.ie and jdconde@mit.edu

S1. Characterization of gold nanoparticles (AuNPs)

S1.1. Characterization of AuNPs with poly(ethylene glycol) (PEG)

Briefly, 10 nM of citrate AuNPs dispersed in aqueous solution (see Methods for AuNPs synthesis) were mixed with 0.006 mg/mL of a commercial hetero-functional (PEG α-Mercapto-ω-carboxy PEG solution, HS-C_2H_4-CONH-PEG-O-C_3H_6-COOH, MW. 3500 Da, Sigma) in an aqueous solution of SDS (0.08%). PEG excess was removed by centrifugation (20.000 ×g, 30 min, 4 ºC) and quantified by the Ellman's Assay as described elsewhere [1,2]. The excess of thiolated chains in the supernatant was quantified by interpolating a calibration curve set by reacting 200 µL of α-Mercapto-ω-carboxy PEG solution in 100 µL of phosphate buffer (0.5 M, pH 7) with 7 µL 5,5'-dithio-bis(2-nitrobenzoic) acid (DTNB, 5 mg/mL) in phosphate buffer (0.5 M, pH 7) and measuring the absorbance at 412 nm (**Figure S1a**) after 10 minutes reaction. The linear range for the PEG chain (**Figure S1b**) obtained by this method is 0–0.1 mg/mL (Abs at 412nm = 6.8991×[PEG, mg/mL] + 0.0588). The number of exchanged chains is given by the difference between the amount determined by this assay and the initial amount incubated with the AuNPs. There is a point at which the nanoparticle becomes saturated with a thiolated layer and is not able to take up more thiolated chains - maximum coverage per gold nanoparticle, i.e. 0.02 mg/mL of PEG (**Figure S1c**). The AuNPs were functionalized with 0.006 mg/mL of PEG corresponding to 30% of PEG saturation on nanoparticle's surface (180.19 ± 7.71 chains per nanoparticle).

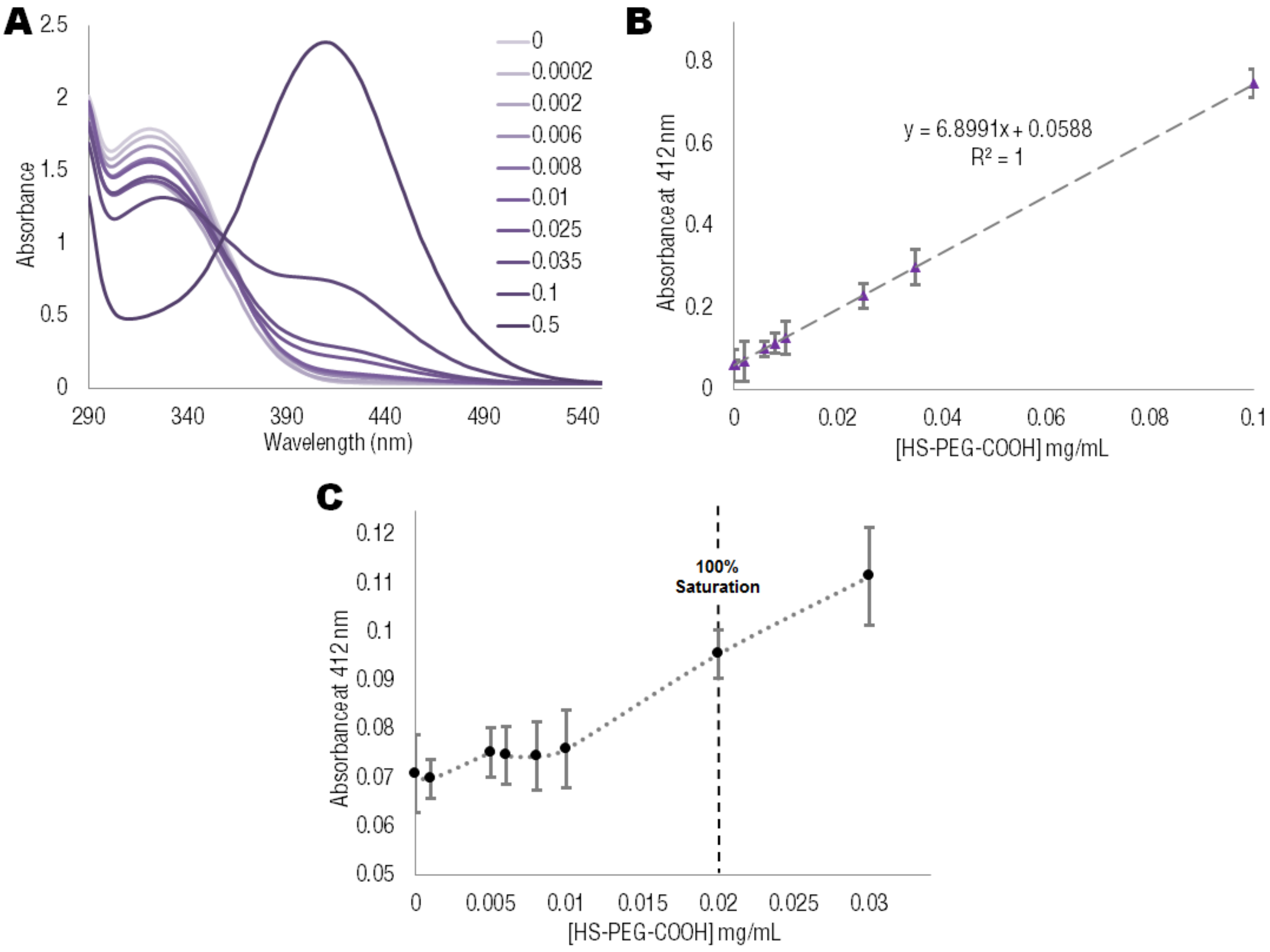

Figure S1. (**A**) Absorbance spectra of DTNB after reaction with increasing amounts (0–0.1 mg/mL) of thiolated PEG. (**B**) Standard calibration curve for PEG chains, whose concentration can be calculated via the following equation: Abs at 412nm = 6.8991×[PEG, mg/mL] + 0.0588, R^2=1. (**C**) Variation of the excess of PEG thiolated chains as a function of the initial concentration in the incubation with 10 nM of AuNPs. The dashed vertical line indicates the 100% saturation, i.e. the PEG concentration above which no more PEG can be bound to the nanoparticle's surface.

S2. Quantification of beacon coverage on AuNP@PEG

Coverage, i.e. average number of labeled beacons per nanoparticle was assessed by the quantification of the excess of the thiolated oligonucleotides (beacons) from the gold nanobeacon synthesis. Oligomers sequences are depicted in **Table S1.** All the three supernatants containing the unbound

oligonucleotides were measured by monitoring the emission spectra of Cy3 (Exc = 550 nm) dye in a microplate reader (Varioskan Flash Multimode Reader, Thermo Scientific). All the AuNPs samples and the standard solutions of the thiol-oligonucleotide beacon were kept at the same pH and ionic strength and calibration for all measurements. Fluorescence emission was converted to molar concentrations of the thiol modified oligonucleotide by interpolation from a standard linear calibration curve. Standard curves were prepared with known concentrations of beacon using the same buffer pH and salt concentrations. The average number of molecular beacon strands per particle was obtained by dividing the oligonucleotide molar concentration by the AuNP concentration.

The total number of thiolated-beacon chains that can be attached per gold nanoparticle are depicted in **Table S2.**

Table S1. Oligomers sequences used in gold nanobeacons assembly.

Oligomers	Sequence and modifications
nanobeacon anti-Kras	Thiol- 5'tttgcatCCTACGCCAC**C**AGCTCCAAatgcaaa 3' -Q705
nanobeacon nonsense	Thiol- 5' tttgcatTTCTCCGAACGTGTCACGTatgcaaa 3'-Q705
Kras target	5'- AGUUGGAGCUGGUGGCGUAGGCA-3'

Note: Kras mutation G12D: GGTAGTTGGAGCTG(**G/A**)TGGCGTAGGCAAGA

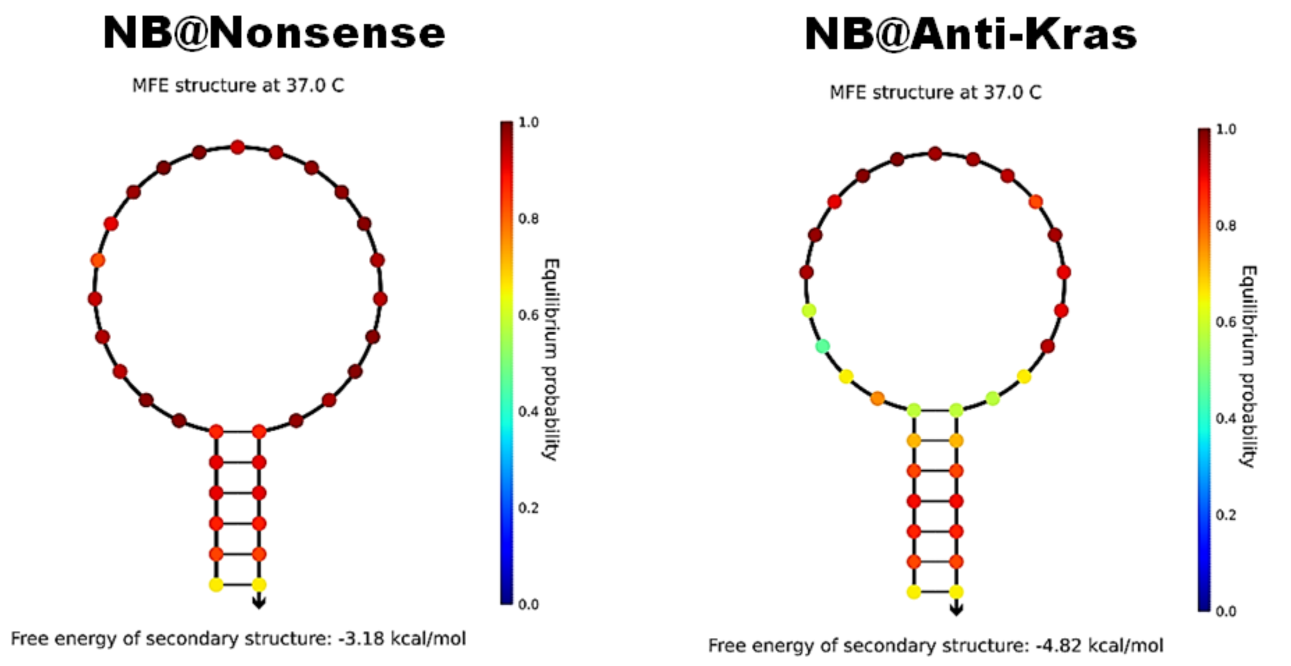

Figure S2. (A) Two-dimensional structures of the sequences for nanobeacons anti-Kras and nonsense at 37ºC, as predicted by NUPACK [3].

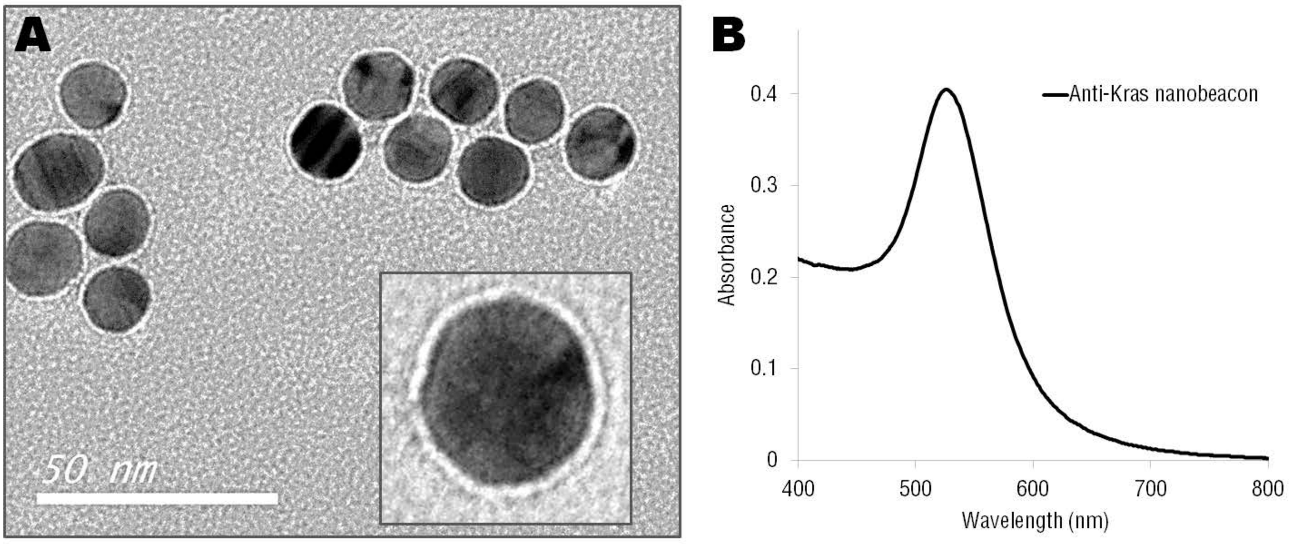

Figure S3. (**A**) Representative TEM images with negative staining of anti-Kras gold nanobeacons. (**B**) UV-Vis spectra of the synthesized anti-Kras nanobeacons, showing the characteristic surface plasmon resonant (SPR) peak at 525 nm.

Table S2. Physico-chemical properties of naked gold nanoparticles (AuNPs) and functionalized gold nanobeacons

Formulation	SPR peak	Size (nm)[a]	Zeta-Potential (mV)[b]	PEG mol per particle	DNA Hairpin mol per particle
Naked AuNPs	520	15.1±1.1	-18.7±2.3	NA	NA
Nanobeacon anti-Kras	525	25.9±3.5	-25.6±1.2	180.19±7.71	45.2±3.1
Nanobeacon nonsense	525	24.7±2.9	-25.3±2.5	180.19±7.71	44.8±2.7

[a] Determined by Dynamic Light Scattering (DLS)
[b] Nanoparticles were analyzed at a concentration of 2 nM in water in a total volume of 1 mL, with 0.1 M KCl, at blood physiological pH (7.4).

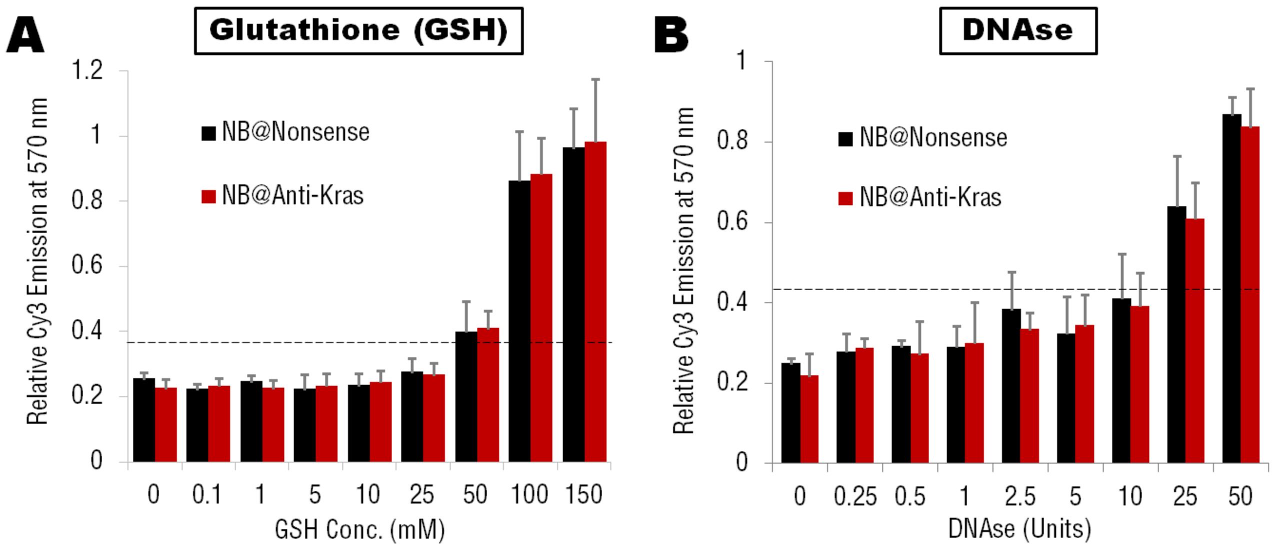

Figure S4. (**A**) Effect of glutathione (GSH) concentration on nanobeacon signal. Stability of gold nanobeacons towards increase concentrations of glutathione (0.1, 1, 5 - physiological concentration, 10, 25, 50, 100 and 150 mM). (**B**) Effect of DNase I concentration on nanobeacon signal. Stability of gold nanobeacons towards increase concentrations of DNase I (0.25, 0.5, 1, 2.5, 5, 10, 25 and 50 Units).

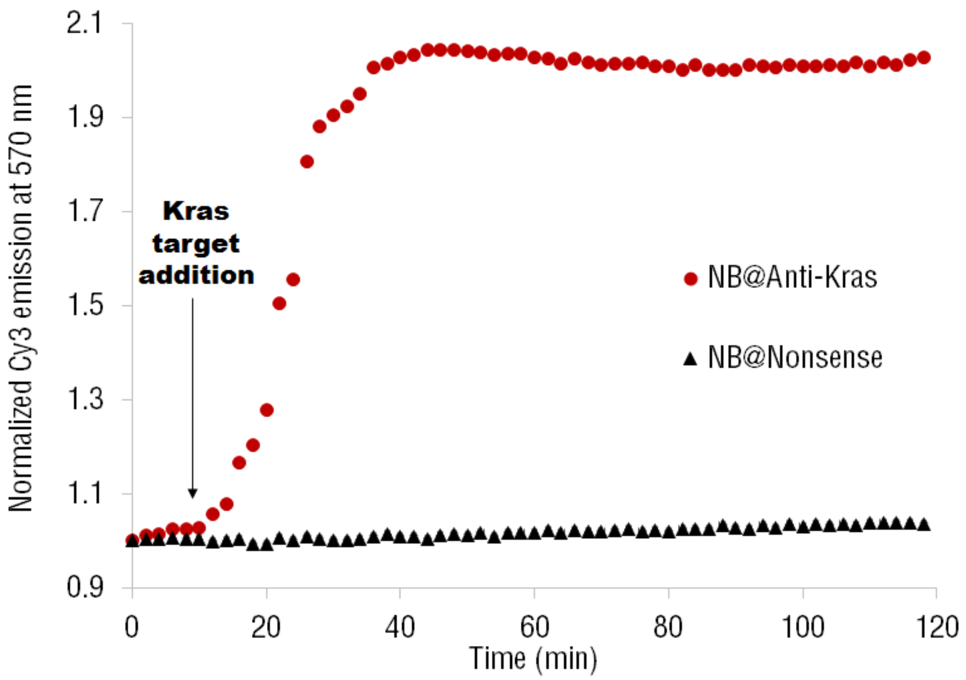

Figure S5. Hybridization kinetics of nonsense and anti-Kras gold nanobeacons at 37º C in presence of 1 µM of the Kras complementary target. Cy3 Exc/Emi = 550/570 nm.

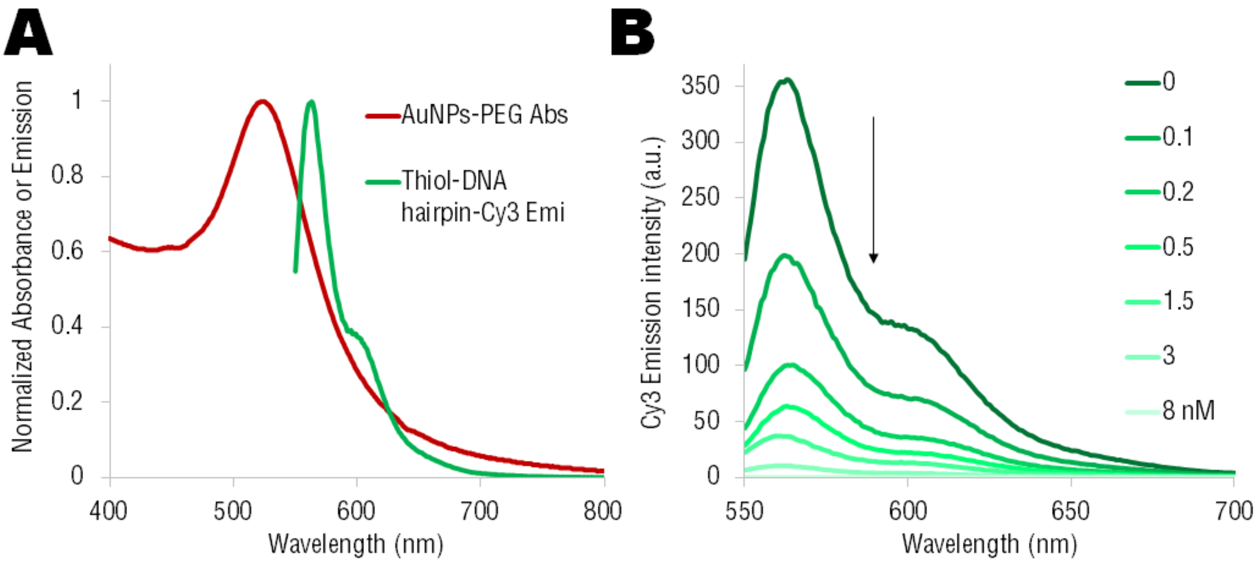

Figure S6. (**A**) Normalized absorbance and emission spectra of AuNPs-PEG and free thiol-DNA hairpin labelled with Cy3 dye. (**B**) Emission intensity change of Cy3 (0.5 µM) after reaction with AuNPs-PEG at different concentration from 0 to 8 nM. Excitation wavelength = 550 nm.

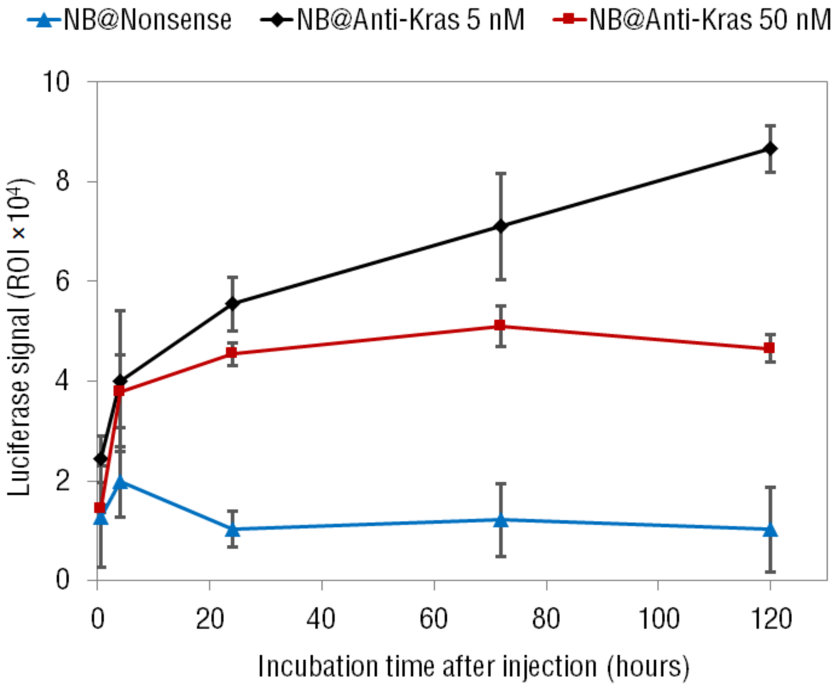

Figure S7. Quantification analysis of tumor ROI as a function of time after treatment with nonsense (50 nM) and anti-Kras nanobeacons (5 and 50 nM).

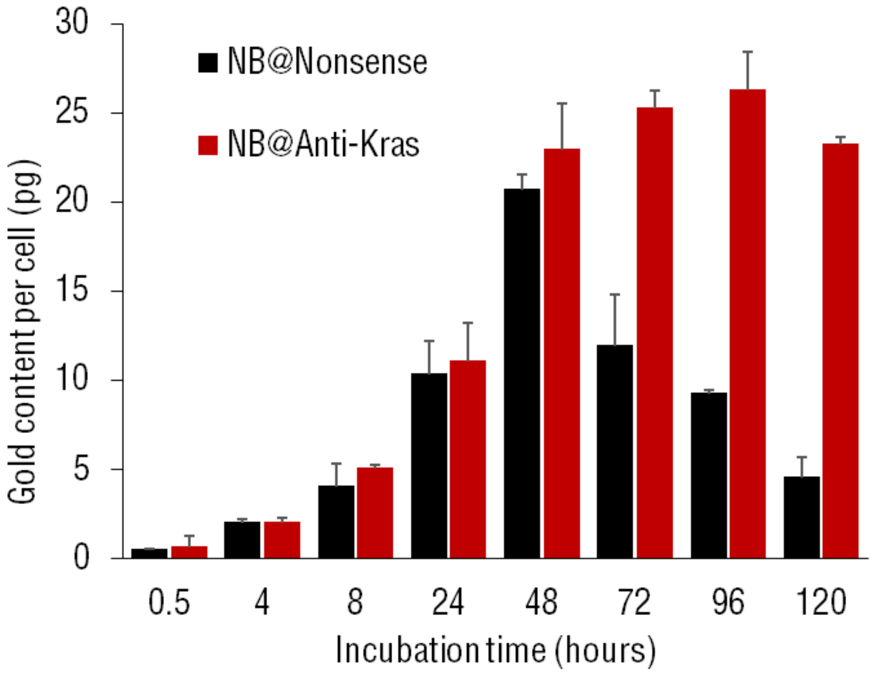

Figure S8. Quantification of gold content in cells by inductively coupled plasma mass spectrometry (ICP-MS) of nonsense and anti-Kras nanobeacons at increasing incubation time points.

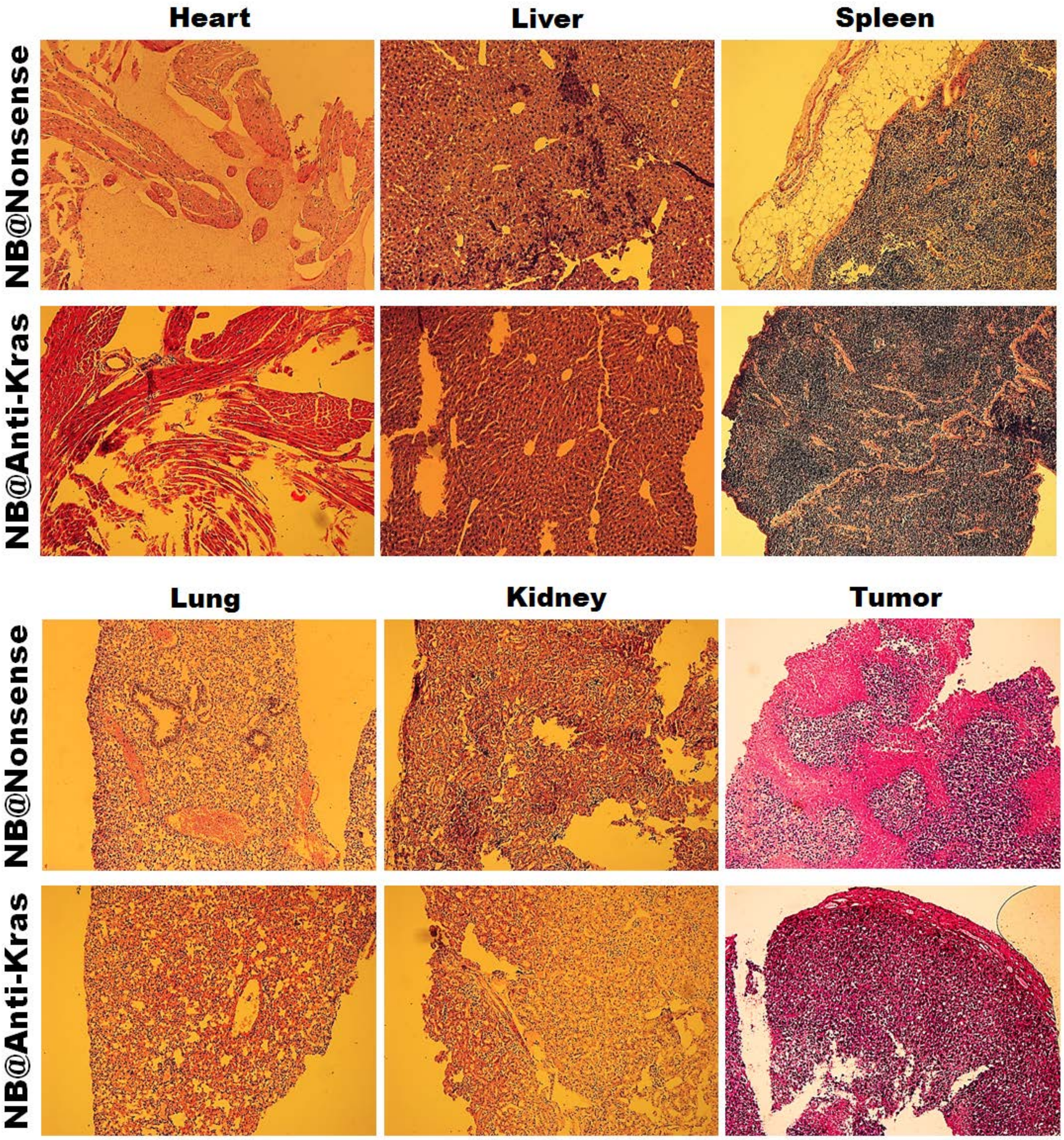

Figure S9. H&E stained tissue sections of the organs (i.e. lung, heart, liver, spleen, lung, kidney and gastric tumor) collected from mice 5 days after intravenous injection of a single dose of nonsense and anti-Kras nanobeacons.

Additional References

1. Sanz, V.; Conde, J.; Hernandez, Y.; Baptista, P. V.; Ibarra, M. R.; de la Fuente, J. M. Effect of PEG biofunctional spacers and TAT peptide on dsRNA loading on gold nanoparticles. *Journal of Nanoparticle Research* **2012,** *14* (6).

2. Conde, J.; Rosa, J.; de la Fuente, J. M.; Baptista, P. V. Gold-nanobeacons for simultaneous gene specific silencing and intracellular tracking of the silencing events. *Biomaterials* **2013,** *34* (10), 2516-2523.

3. Zadeh, J. N.; Steenberg, C. D.; Bois, J. S.; Wolfe, B. R.; Pierce, M. B.; Khan, A. R.; Dirks, R. M.; Pierce, N. A. NUPACK: Analysis and design of nucleic acid systems. *J. Comput. Chem.* **2011,** *32* (1), 170-173.
